# Aligning enterprise viability with population health: a public health–oriented food entrepreneurship framework for urban food systems

**DOI:** 10.3389/fpubh.2026.1963621

**Published:** 2026-10-26

**Authors:** M. Ajay Adithya, R. Geetha, S. Sundar, Palanivel R. Velmurugan

**Affiliations:** 1 Department of Commerce, School of Social Sciences and Languages, Vellore Institute of Technology, Vellore, Tamil Nadu, India; 2 Department of Commerce, Faculty of Management, Alagappa University, Karaikudi, Tamil Nadu, India; 3 Faculty of Economics and Business Administration, Berlin School of Business and Innovation Gesellschaft mit beschränkter Haftung (GmbH), Berlin, Germany

**Keywords:** food entrepreneurship, food system, population health, public health, urban

## 1 Introduction: the overlooked public-health role of micro-food enterprises

In addition to supermarkets and known restaurants, street food vendors, food trucks and kiosks, market stalls and other small food-service enterprises shape urban food environments. The micro- and mobile-fodder businesses are part of the familiar environments in which food is bought and eaten. Therefore, product and price decisions, operating locations, food preparation practices, and marketing decisions can have an impact on the safety, accessibility, affordability, and nutrition of local food environments (1, 2).

Traditionally, public health control of small food businesses has focused on hygiene, sanitation, licensing, inspection and the prevention of food-borne illness (3, 4). While such functions are still important, a risk and compliance-driven emphasis of the relationship between food entrepreneurship and population health only addresses part of the relationship. Micro-food enterprises are at the same time commercial actors and local food system actors. Food safety, nutritional attributes, food accessibility and environmental attributes should therefore work within a business which has to control cost, customer demand, pricing, competitive pressures, food supply reliability and continuity.

This Opinion suggests that the Public Health–Oriented Food Entrepreneurship (PHOFE) can be used as a conceptual tool to explore this intersection. In action, PHOFE links the viability of enterprises to the population-health goals along five interconnections: food safety and quality; nutritional value; economic and spatial accessibility; commercial sustainability; environmental responsibility; and an enabling governance and institutional ecosystem. It is not as if entrepreneurship is a health-promoting phenomenon by itself and that business is a replacement for public-health institutions.

On this basis, the Opinion outlines the PHOFE framework, explores the commercial–public health alignment problem and suggests entrepreneurial public-health governance as a way to bring together enterprise viability and healthier and more equitable urban food systems.

## 2 Beyond compliance: micro-food enterprises as actors in urban food environments

Traditionally, public health monitoring of micro-food enterprises has focused on licensing, hygienic requirements, sanitation, food-handlers' requirements, monitoring and food-safety compliance (3, 4). These functions are all important aspects of consumer protection, but they have not attempted to reflect the wider role that small food businesses play in urban food environments. The food environment, which is shaped by food availability, food costs and food promotion, is also a factor in these environments (1, 5).

Everyday business decisions can thus assume public-health relevance. The design of a menu can have an impact on the food options provided to consumers, the pricing of food can impact the economic accessibility of the food to consumers, and location of food can have an impact on which populations are able to easily access food (1, 5, 6). Procurement, storage and preparation processes impact food quality and safety as well as marketing efforts can contribute to the visibility and attractiveness of specific options (3, 5). Other city locations where mobile companies can serve include workplaces, educational institutions, transport hubs, residential areas and more.

But, it cannot be taken for granted that micro-food entrepreneurship is per se health promoting. Low cost, tastey products may be preferred for commercial reasons over more nutritious options, and resource and capability limitations may limit investment in infrastructure, training, safer technologies or product reformulation (7). Simply having mobility does not mean that everyone has the same opportunities for space, and a low cost does not mean that the food is nutritious (1, 5). Commercial, regulatory, infrastructural and socioeconomic conditions, in which companies function, therefore, partially affect the public-health value.

The significant change is therefore moving from a compliance paradigm to an alignment paradigm. In addition to compliance requirements, alignment also asks about the broader entrepreneurial context and whether “commercial decisions” can be made “compatible with population-health objectives”. This separation is the starting point for thinking of micro-food enterprises as not only regulated economic enterprises but potentially as actors in the urban food system.

## 3 Public health–oriented food entrepreneurship: a proposed framework

To bring the public-health value of micro food enterprises together in a coherent conceptual approach, we suggest the Public Health–Oriented Food Entrepreneurship (PHOFE) framework. PHOFE views food entrepreneurship as an enterprise activity that intentionally integrates enterprise value with public health values. It includes five dimensions: food safety and food quality; nutrition; economic and spatial access; commercial sustainability; and food environment, which are all interconnected and underpinned by a governance and institutional framework.

Food safety and quality is the basic dimension. Minimum conditions for broader public-health value are set by hygiene, safe preparation and storage, sanitation, competence of food-handlers, quality assurance and regulatory compliance (3, 4). Nutritional value applies to what foods are included and how they are presented, and include changes that are feasible at the ingredient and preparation level, as well as portion size, menu planning and availability of nutritionally better options (5). Food access is linked to both economic and spatial accessibility, which is to say, accessibility depends on food pricing and on the availability of the food provided by the operating location (1, 5, 6).

Commercial sustainability appreciates that health-related practices are not likely to be sustainable in the long term if they aren't being undertaken in a way that can be sustained operationally and financially. Thus, conditions for implementing are not the health outcomes, but cost management, customer demand, cash flow, supply reliability, entrepreneurial ability, and business continuity. Environmental responsibility is an awareness that the food and packaging waste, resource use, procurement and waste disposal may have repercussions beyond the enterprise (8, 9).

These dimensions may be made observable in domains that can be used for empirical and policy application. Food safety and quality can be measured by compliance, hygiene, food-handling competence and storage; nutritional value by food composition and healthier food; food accessibility by food price, location, and access to undervalued groups of people; food commercial sustainability by continuity, food costing, and food supply; and food environmental sustainability by waste, packaging, resource consumption, and procurement. Governance conditions can be analyzed individually via access to infrastructure, finance, training, digital systems, incentives, and regulatory support. Future research is needed to decide which indicators and thresholds are appropriate for different contexts, not all dimensions are equally important across contexts.

PHOFE suggests that aligning all of these dimensions could help to enable safer, healthier, accessible, commercially viable and environmentally sustainable food provision as shown in Figure 1. These are pathways rather than causal pathways, and must be empirically tested.

**Figure 1:**
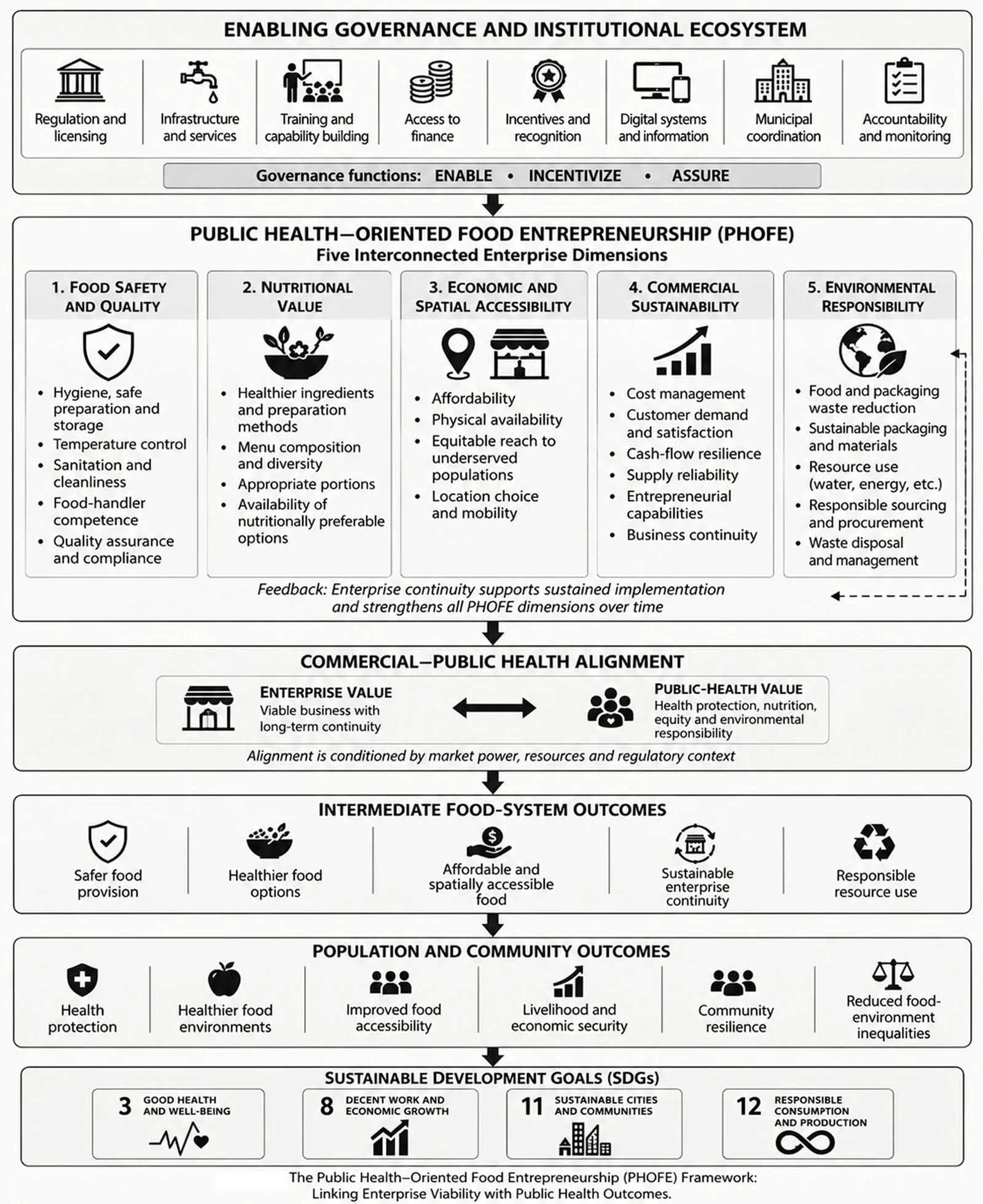
The Public Health-Oriented Food Entrepreneurship (PHOFE) framework: linking enterprise viablity with public health outcomes. Authors' conceptualization. The visual rendering of the figure was created with the assistance of OpenAI's ChatGPT image-generation tool (GPT-Image-1.5) and reviewed by the authors. No third-party images or previously published graphical material were used.

## 4 The commercial–public health alignment problem

The PHOFE framework is based on a fundamental conflict—commercial priorities and public health objectives can conflict and do not necessarily align. Micro-food business owners are required to attend to customers' orders while keeping expenses, price, competition, supply reliability, and income in check. All of these investments are necessary to achieve public-health goals at the same time. To achieve these goals for resource-constrained businesses may involve financial and operational compromises (2, 10).

This tension is referred to as the commercial–public health alignment problem. However, the use of nutritionally more desirable ingredients may add to the cost of production in a price sensitive market (6), and investment in safer equipment, improved storage, sustainable packaging, or employee training may require extra operating costs, capital, and/or knowledge (2, 10). On the other hand, short-term strategies focusing on low prices, convenience, demand, or short-term margins may not have an impact on the nutritional quality, safety, accessibility, or environmental performance.

Political economy of food systems also influences these trade-offs. While commercial determinants of health aren't always obvious, they can work within systems where private-sector practices, market structures and unequal distributions of resources and power have a negative effect on population health (11, 12). Small-scale food businesses may have significantly reduced leverage over locations, access to finance, procurement, regulatory representation and control over commercially advantageous locations compared to larger businesses. Therefore, the blame for healthier food environments should not be leveled exclusively at the individual entrepreneur; the market power, resources, and regulatory burden distributions influence the ability of the entrepreneur to help bring commercial and public health objectives to line up.

The dual-value creation is therefore treated as an organizing principle for PHOFE: enterprise value is defined by the viable and sustainable organization for the continuity of business and public-health value as the focal point of the commercial operation for the creation of safer, nutritionally responsible, accessible and environmentally sustainable food environments. Commercial sustainability is thus a condition for implementation and not a goal to achieve. Essential public-health protections must not be sacrificed to enterprise viability; practices that are not sustainable are not likely to produce long-lasting public-health value. The puzzle here is to develop a policy framework that enables these kinds of values to complement each other rather than simply assuming that they do.

## 5 From regulatory control to entrepreneurial public-health governance

Governance that goes beyond inspection and enforcement, yet maintains critical food-safety protections, is needed to tackle the commercial–public health alignment challenge. We suggest entrepreneurial public-health governance as a strategy that involves creating regulatory frameworks alongside institutional factors that facilitate micro-food businesses to learn and maintain health-focused practices.

The following are three complementary functions that can be built around this approach: enable, incentivize and assure. Clear registration processes, accessible food-safety and nutrition information, entrepreneurial training and adequate digital systems are all opportunities for public institutions to facilitate responsible enterprise. There are opportunities for public institutions to facilitate responsible enterprise through clear processes of registration, accessible guidance on food-safety and nutrition, entrepreneurial training, and adequate digital systems. Hygiene and environmental requirements can be realistically met or not if clean water, sanitation, electricity, and space for safe operations and waste disposal are available (10, 13, 14). For individuals where it is not feasible to provide individual vending, designated vending spaces and shared facilities can assist in integrating the commercial operation with necessary public-health facilities.

Second, governance can encourage practices which go beyond the minimum. Nutritional and environmental goals can be integrated into enterprises through business-development services as well as the provision of appropriate finance, supply-chain management and innovation support (2). However, incentives should be clearly stated and tied to visible criteria, and not to "symbolic recognition.

Thirdly, authorities have to ensure fundamental safeguards. Food-safety standards must not be watered down due to administrative simplification; licensing, inspection, monitoring and accountability still needed, especially if unsafe practices pose a threat to population-health (3, 4).

These functions need cross-sector co-ordination. The need for food regulation, infrastructure, financing, urban space and enterprise development crosses institutional boundaries, explaining the complementary role that may be played by municipal authorities, public-health and food-safety agencies, urban planners, entrepreneurship-support organizations, financial institutions, entrepreneur associations and communities (15). Local food preferences, purchasing power and consumption patterns are particularly relevant in community participation where interventions need to address these needs (2).

The entrepreneurial governance of public health is therefore not linear, and does not consist of a relationship between a regulator and a regulated entity, but rather a relationship between a public authority, an entrepreneur and a community. Its goal is to facilitate the viability of responsible entrepreneurship without deregulating or giving unconditional support to business. Its agenda is not to deregulate or to give unconditional support to business, but rather to make it more viable to be a responsible entrepreneur and to ensure accountability and enforceable public-health protections.

## 6 Discussion: from regulated vendors to public-health partners

A key contribution of PHOFE is to situate enterprise viability as a requirement for the implementation of public-health oriented food systems, but not as an end goal beyond public health. The framework thus points to a question that can only be addressed by commercial, institutional and governance conditions so that health-oriented practices can be made feasible and sustainable in micro food enterprises.

This is an equity related question. Having more micro food enterprises doesn't always lead to equitable access to healthier food. Exposure and access in local food environments may be influenced by socioeconomic inequalities (16, 17) and/or commercial incentives may favor sites that have more purchasing power or customer foot traffic. Meanwhile, mandates designed to create better food environments may have a disproportionate impact on low-resource entrepreneurs. Therefore, an equity-based approach to PHOFE should take into account both consumers and entrepreneurs—who is accessing safer and healthier food, who is not, and who is taking the financial and regulatory burden of achieving public-health goals.

PHOFE should be viewed as an idea and a policy rather than an empirically tested causal model. Future research is needed to operationalize the five enterprise dimensions and governance conditions, to validate the proposed indicators in terms of their reliability and contextual appropriateness, and to validate their relationship with food safety, food nutritional quality, food prices, spatial accessibility, enterprise continuity, and food-environment equity. Comparative studies of cities and countries may further establish whether the importances of the PHOFE dimensions are relative to the regulatory framework, the market structure and the socio-economic context.

The framework is most closely related to SDG 3 (Good Health and Well Being), but there are also linkages to SDG 8 (Decent Work and Economic Growth), SDG 11 (Sustainable Cities and Communities) and SDG 12 (Responsible Consumption and Production) with a focus on livelihoods, urban food environments and responsible resource use (18–21).

Finally, the micro-food entrepreneur is not a public health agent by default, but can't simply be seen as a liability for regulatory authorities. They can play a role in providing healthier food environments, but it relies on the context of their work. The issue to which cities must respond is not only whether micro-food enterprises can operate safely but how entrepreneurial ecosystems can ensure that food provision is safer, healthier, more equitable and environmentally friendly – and commercially sustainable.
